# Identification of intraoperative hypoxemia and hypoproteinemia as prognostic indicators in anastomotic leakage post-radical gastrectomy: an 8-year multicenter study utilizing machine learning techniques

**DOI:** 10.3389/fonc.2024.1471137

**Published:** 2024-11-27

**Authors:** Yuan Liu, Songyun Zhao, Xingchen Shang, Wei Shen, Wenyi Du, Ning Zhou

**Affiliations:** ^1^ Department of General Surgery, Wuxi People’s Hospital Affiliated to Nanjing Medical University, Wuxi, China; ^2^ Department of Neurosurgery, Wuxi People’s Hospital Affiliated to Nanjing Medical University, Wuxi, China

**Keywords:** gastric tumor, gastrectomy, anastomotic leakage, prognosis, risk factor, machine learning

## Abstract

**Background:**

Complications and mortality rates following gastrectomy for gastric cancer have improved over recent years; however, complications such as anastomotic leakage (AL) continue to significantly impact both immediate and long-term prognoses. This study aimed to develop a machine learning model to identify preoperative and intraoperative high-risk factors and predict mortality in patients with AL after radical gastrectomy.

**Methods:**

For this investigation, 906 patients diagnosed with gastric cancer were enrolled and evaluated, with a comprehensive set of 36 feature variables collected. We employed three distinct machine learning algorithms—extreme gradient boosting (XGBoost), random forest (RF), and k-nearest neighbor (KNN)—to develop our models. To ensure model robustness, we applied k-fold cross-validation for internal validation of the four models and subsequently validated them using independent datasets.

**Results:**

In contrast to the other machine learning models employed in this study, the XGBoost algorithm exhibited superior predictive performance in identifying mortality risk factors for patients with AL across one, three, and five-year intervals. The analysis identified several common risk factors affecting mortality rates at these intervals, including advanced age, hypoproteinemia, a history of anemia and hypertension, prolonged operative time, increased intraoperative bleeding, low intraoperative percutaneous arterial oxygen saturation (SPO_2_) levels, T3 and T4 tumors, tumor lymph node invasion, and tumor peripheral nerve invasion (PNI).

**Conclusion:**

Among the three machine learning models examined in this study, the XGBoost algorithm exhibited superior predictive capabilities concerning the prognosis of patients with AL following gastrectomy. Additionally, the use of machine learning models offers valuable assistance to clinicians in identifying crucial prognostic factors, thereby enhancing personalized patient monitoring and management.

## Introduction

Among malignant tumors, gastric cancer ranks second only to lung cancer in incidence, and its pervasive and severe nature renders it a major global public health concern. Despite considerable advancements in early detection and treatment through technological progress, gastric cancer remains a formidable threat to patient health due to its insidious and complex characteristics (1, 2). Consequently, surgery remains the principal modality for the curative treatment of this malignancy (3). Recent advancements have seen a shift from traditional open surgical approaches to minimally invasive laparoscopic and robotic techniques, leading to enhanced prognostic outcomes, reduced intraoperative trauma, and expedited postoperative recovery (4). Despite these improvements, the complex anatomical structure of the stomach, along with the distribution of adjacent lymph nodes and the technical challenges inherent in gastrectomy, often exposes patients to increased risks of severe postoperative complications such as anastomotic leakage (AL) and venous embolism (5, 6). Among the potential complications following cancer surgery, postoperative AL is particularly severe, with the potential to cause noncancer-related mortality. Additionally, AL increases the risk of subsequent complications, including anastomotic stricture and abdominal infection, which necessitate further medical interventions such as additional surgery, drainage, and antibiotic therapy. These additional medical costs can impose a significant burden on both patients and the healthcare system (7, 8).

Machine learning (ML) is a computational technique specializing in empirical prediction and pattern recognition from complex, multidimensional datasets. It leverages algorithms and statistical models to analyze vast quantities of data, uncovering latent patterns and relationships to facilitate accurate predictions and informed decisions. Recent advancements in machine learning have markedly expanded its utility in contemporary medical research. By processing intricate medical data—such as genomic sequences, medical imaging, and electronic health records—machine learning enables researchers to elucidate disease mechanisms, predict disease risk, tailor personalized treatment strategies, and enhance the precision and efficiency of clinical decision-making. The ongoing evolution of this technology increasingly augments its potential in the medical domain, offering robust support for early disease detection, treatment assessment, and novel drug development (9–11). Nonetheless, there remains a paucity of studies on machine learning models to prognosticate patients with AL following gastrectomy. Therefore, it is imperative to develop and compare prediction models based on various machine learning strategies to facilitate personalized and intelligent treatment and monitoring for postoperative patients with AL.

## Materials and methods

### Study Subjects

In this study, data were sourced from the clinical databases of the Affiliated Wuxi People’s Hospital of Nanjing Medical University, Wuxi Second People’s Hospital, and Shandong Provincial Hospital affiliated with Shandong First Medical University. The inclusion criteria were: (1) patients who underwent either laparoscopic-assisted or traditional open radical gastrectomy; (2) surgical teams composed of senior surgeons adept in performing radical gastrectomy; and (3) patients diagnosed with anastomotic leakage (AL). Exclusion criteria included: (1) patients with concurrent malignant tumors; (2) patients with confirmed distant metastasis of gastric cancer via pathological examination or imaging; (3) patients with severe cardiovascular or respiratory conditions; (4) patients with significant organ dysfunction, such as liver or kidney disease; and (5) patients with incomplete case, clinical data, or follow-up information. All participants were monitored for a minimum of five years post-surgery. The study was approved by the ethics committees of the Affiliated Wuxi People’s Hospital of Nanjing Medical University, Wuxi Second People’s Hospital, and Shandong Provincial Hospital affiliated with Shandong First Medical University, under approval number KY22086.

### Diagnosis of AL and determination of associated factors

The diagnosis of AL in the present study adhered to the definition established by the Esophagectomy Complications Consensus Group (ECCG) (12). AL was diagnosed based on the following criteria: (1) a marked rise in the patient’s temperature following its normalization after surgery or sustained fever; (2) an increase in the leukocyte count and neutrophil ratio; (3) clinical signs of peritoneal irritation; (4) edema at the anastomotic site with observable exudate, confirmed by CT imaging; and (5) the presence of blue-colored drainage fluid in the drainage tube following methylene blue infusion. The patient met all five criteria, confirming the diagnosis of AL.

### Study design and data collection

Demographic characteristics, fundamental clinical features, medical history, preoperative and postoperative laboratory indices, as well as tumor and intraoperative attributes of patients, were assessed for clinical information. Preoperative laboratory tests, including albumin (ALB), carcinoembryonic antigen (CEA), and carbohydrate antigen 19-9 (CA19-9), were obtained within 24 hours prior to surgery. Postoperative laboratory tests, comprising procalcitonin (PCT), C-reactive protein (CRP), and serum amyloid A (SAA), were collected within 48 hours following surgery. Patient demographics included sex, age, BMI, smoking history, and alcohol consumption history. Basic clinical characteristics evaluated encompassed the American Society of Anesthesiologists (ASA) score, Nutrition Risk Screening 2002 (NRS2002) score, history of previous surgeries, adjuvant chemotherapy, and adjuvant radiotherapy. Medical history information included anemia, diabetes mellitus, hypertension, chronic obstructive pulmonary disease (COPD), hyperlipidemia, and coronary heart disease (CHD). Tumor characteristics examined included tumor T-stage, N-stage, peripheral nerve invasion (PNI), tumor size, and tumor count. Intraoperative variables recorded included the type of procedure, anastomosis method and type, procedure duration, intraoperative bleeding, blood transfusion, percutaneous arterial oxygen saturation (SPO_2_) status, and whether the procedure was emergent. Outcome measures for this study included patient mortality at one, three, and five years.

### Statistical analysis

Continuous variables were presented as medians with interquartile ranges (Q1-Q3), while categorical variables were reported as frequencies and percentages. The chi-square test was used to compare categorical variables between groups, and the t test was applied to continuous variables meeting the normality assumption. For continuous variables that did not follow a normal distribution, the rank sum test was utilized. Statistical significance was defined as a two-sided P value of less than 0.05. Statistical analyses were conducted using SPSS, R, and Python software.

### Establishment and evaluation of predictive models for machine learning algorithms

(1) Data preprocessing: Patients diagnosed with gastric cancer at Wuxi People’s Hospital and Wuxi Second People’s Hospital from January 2010 to January 2018 were designated as the internal validation set, while patients from Shandong Provincial Hospital affiliated with Shandong First Medical University during the same period constituted the external validation set. The internal validation set was randomly partitioned into a training set (70%) and a test set (30%). (2) Data from the internal validation set underwent univariate analysis, with significant variables selected for the subsequent prediction model construction. (3) Build and evaluate prediction models: The selected feature variables were incorporated into prediction models utilizing three machine learning algorithms: extreme gradient boosting (XGBoost), random forest (RF), and k-nearest neighbor (KNN). K-fold cross-validation was employed to compare and select the optimal model algorithms, given its straightforward implementation and reduced bias compared to alternative methods. Hyperparameters were fine-tuned using grid search, with k-fold cross-validation conducted on the internal validation set using a resampling method with k=5. The k-fold cross-validation procedure was as follows: the dataset was divided into five subsets, one of which served as the test set while the remaining subsets constituted the training set. The model was trained and hyperparameters were adjusted using the training set, and performance was assessed using the test set. This process was iterated until each subset was used as a test set. Model evaluation metrics, including area under the curve (AUC), accuracy, sensitivity, and specificity, were recorded and averaged across the k iterations to provide a comprehensive estimate of model performance. We further assessed the models for their ability to predict the three outcome indicators by examining their discrimination, calibration, and clinical utility. The best model was selected for prediction analysis. We plotted receiver operating characteristic (ROC) curves to derive area under the curve (AUC) values and assess the model’s predictive performance. Calibration curves were also plotted to compare predicted outcomes with actual results. Additionally, decision curve analysis (DCA) was performed to evaluate whether model-based decisions were beneficial to patients. The DCA curve begins at the intersection of the red curve with the “All” curve and concludes at the intersection of the red curve with the “None” curve, within which patient benefit is indicated. (4) The optimal model was validated using an external test set, with ROC curves plotted to evaluate its generalizability and predictive accuracy. (5) Model interpretation: The influence of each feature on predictions was analyzed using SHAP (Shapley Additive Explanations) analysis, which calculates Shapley values. These values were utilized to create SHAP summary plots, facilitating the ranking of risk factor importance.

## Results

### Clinical information of the patients

**Figure 1:**
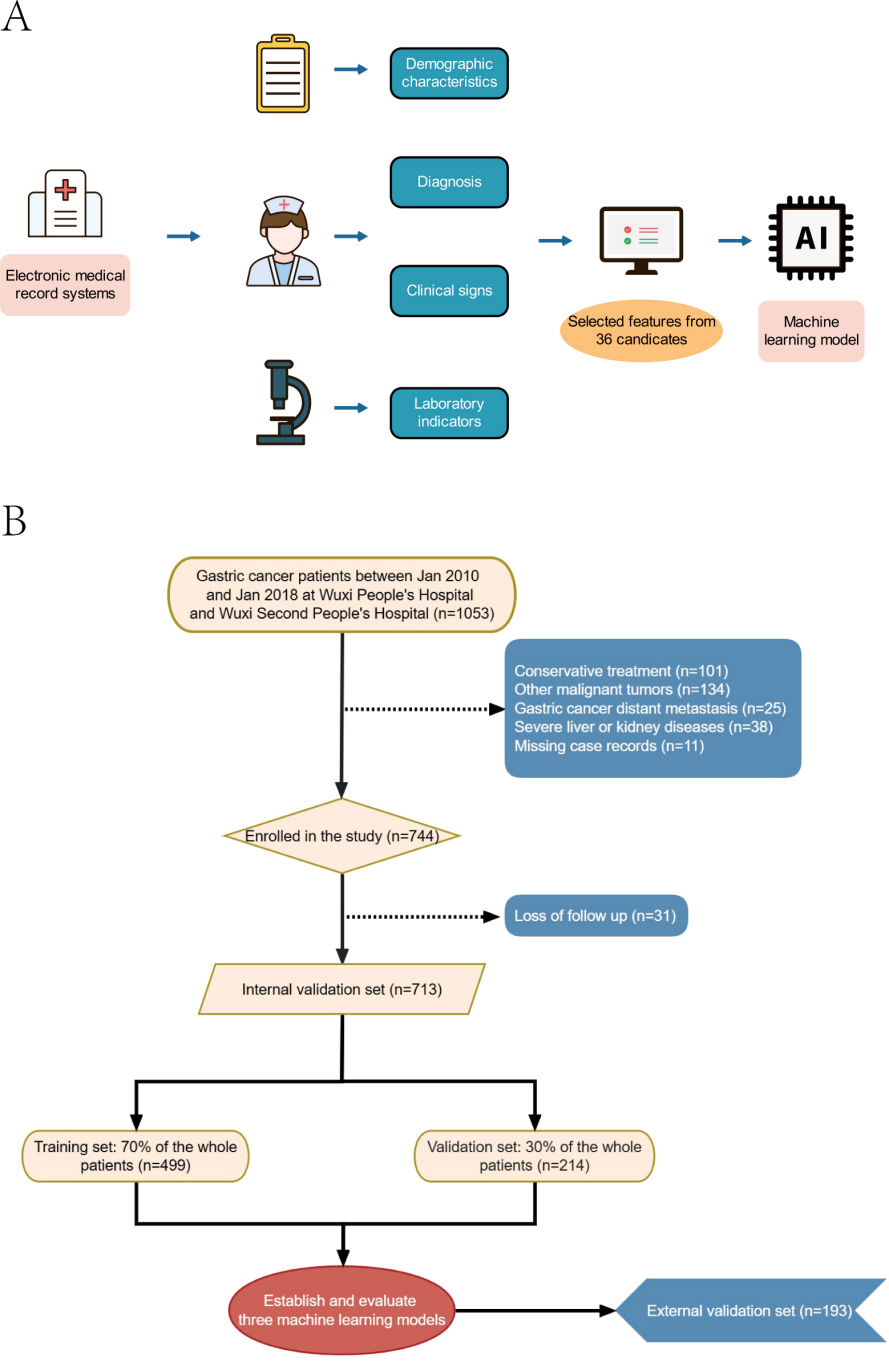
Model-making process and flowchart of the study. **(A)** Study design flow chart. **(B)** Flow diagram of patients included in the study.

The study encompassed a total of 906 patients, of whom 86 (9.49%) succumbed within one year, 270 (29.8%) within three years, and 366 (40.4%) within five years (**Figures 1A, B**). Within this cohort, 713 patients with gastric cancer constituted the internal validation set, with 66 (9.26%) one-year deaths, 206 (28.89%) three-year deaths, and 281 (39.41%) five-year deaths. The external validation set comprised 193 gastric cancer patients, of whom 20 (10.36%) died within one year, 64 (33.16%) within three years, and 85 (44.04%) within five years. The original data presented in the study are detailed in **Supplementary Table S1**.

### Screening for risk factors for death at one, three and five years in patients with AL

The findings from univariate analysis demonstrated that certain factors independently influenced one-year mortality in patients with AL. These factors included age, albumin levels, NRS2002 score, history of anemia and hypertension, emergency surgery, operative time, intraoperative bleeding and SPO_2_ levels, tumor T stage, tumor lymph node invasion, and tumor PNI (P<0.05). Similarly, for three-year mortality in patients with AL, age, ALB levels, history of anemia and hypertension, time of surgery, intraoperative bleeding, blood transfusion, intraoperative SPO_2_ levels, tumor T stage, tumor lymph node invasion, and tumor PNI were found to be significant independent influencing factors. In addition, sex, age, ALB levels, history of anemia and hypertension, surgical approach, operative time, intraoperative bleeding and blood transfusion, intraoperative SPO_2_ levels, tumor size, tumor T-stage, tumor lymph node invasion, and tumor PNI were found to significantly influence five-year mortality in patients with AL (**Table 1**). We additionally compared the baseline characteristics of the internal validation set with those of the external validation set, as well as the training set with the test set, identifying differences in several aspects. These findings further underscore the model’s generalizability and its potential applicability across a wider range of clinical scenarios (**Supplementary Tables S2**, **S3**).

**Table 1:** Univariate analysis of variables related to postoperative prognosis.

Variables		One-year mortality		Three-year mortality		Five-year mortality	
		OR(95%CI)	P value	OR(95%CI)	P value	OR(95%CI)	P value
Sex	Female	Reference		Reference		Reference	
	Male	1.024[0.614,1.705]	0.929	0.73[0.528,1.011]	0.058	0.564[0.416,0.764]	<0.001
Age	<65	Reference		Reference		Reference	
	≥65	3.365[1.999,5.665]	<0.001	4.098[2.911,5.769]	<0.001	9.438[6.605,13.485]	<0.001
BMI	<25 kg/m^2^	Reference		Reference		Reference	
	≥25 kg/m^2^	1.057[0.604,1.850]	0.846	1.136[0.795,1.625]	0.484	1.189[0.852,1.660]	0.308
ASA	<3	Reference		Reference		Reference	
	≥3	1.165[0.676,2.011]	0.582	0.951[0.664,1.361]	0.782	0.939[0.674,1.310]	0.712
Drinking history	No	Reference		Reference		Reference	
	Yes	1.036[0.586,1.830]	0.904	0.698[0.476,1.023]	0.065	0.837[0.593,1.179]	0.308
Smoking history	No	Reference		Reference		Reference	
	Yes	1.623[0.963,2.737]	0.069	1.379[0.974,1.951]	0.07	1.353[0.977,1.875]	0.069
ALB	≥30 g/L	Reference		Reference		Reference	
	<30 g/L	2.047[1.230,3.407]	0.006	4.045[2.877,5.687]	<0.001	6.274[4.489,8.769]	<0.001
NRS2002 score	<3	Reference		Reference		Reference	
	≥3	2.34[1.367,4.006]	0.002	1.339[0.912,1.967]	0.137	1.297[0.902,1.865]	0.161
Surgical history	No	Reference		Reference		Reference	
	Yes	1.463[0.824,2.599]	0.194	1.159[0.784,1.714]	0.458	1.146[0.795,1.652]	0.465
Anemia	No	Reference		Reference		Reference	
	Yes	2.579[1.538,4.324]	<0.001	4.204[2.945,6.002]	<0.001	8.572[5.834,12.597]	<0.001
Hyperlipidemia	No	Reference		Reference		Reference	
	Yes	1.435[0.789,2.607]	0.236	0.968[0.639,1.468]	0.88	0.932[0.634,1.372]	0.722
Hypertension	No	Reference		Reference		Reference	
	Yes	1.73[1.041,2.876]	0.035	3.042[2.177,4.250]	<0.001	4.196[3.040,5.791]	<0.001
Diabetes	No	Reference		Reference		Reference	
	Yes	1.436[0.779,2.646]	0.246	1.308[0.866,1.976]	0.202	1.259[0.852,1.859]	0.248
COPD	No	Reference		Reference		Reference	
	Yes	1.807[0.847,3.855]	0.126	1.551[0.899,2.676]	0.115	1.438[0.850,2.436]	0.176
CHD	No	Reference		Reference		Reference	
	Yes	1.993[0.989,4.016]	0.054	1.322[0.784,2.228]	0.296	1.097[0.665,1.812]	0.716
Adjuvant Radiotherapy	No	Reference		Reference		Reference	
	Yes	1.323[0.729,2.400]	0.357	1.181[0.794,1.758]	0.411	1.202[0.828,1.744]	0.334
Adjuvant Chemotherapy	No	Reference		Reference		Reference	
	Yes	0.84[0.453,1.557]	0.58	1.142[0.785,1.660]	0.487	1.087[0.766,1.543]	0.64
Surgical procedure	Laparoscopic surgery	Reference		Reference		Reference	
	Open surgery	1.19[0.706,2.003]	0.514	0.674[0.486,0.934]	0.018	0.681[0.502,0.923]	0.013
Emergency surgery	No	Reference		Reference		Reference	
	Yes	1.685[1.013,2.800]	0.044	1.054[0.756,1.470]	0.755	1.078[0.792,1.467]	0.635
Surgery type	Proximal gastrectomy	Reference		Reference		Reference	
	Distal gastrectomy	1.038[0.538,2.001]	0.911	0.974[0.657,1.444]	0.895	0.916[0.634,1.322]	0.639
	Total gastrectomy	1.567[0.837,2.934]	0.16	1.021[0.682,1.530]	0.918	1.156[0.796,1.681]	0.446
Anastomosis method	Anastomosis instruments	Reference		Reference		Reference	
	Manual anastomosis	1.716[0.996,2.956]	0.052	1.27[0.875,1.844]	0.209	1.245[0.877,1.768]	0.221
Anastomosis type	Billroth I	Reference		Reference		Reference	
	Billroth II	1.265[0.675,2.369]	0.463	1.297[0.860,1.955]	0.214	1.156[0.789,1.694]	0.456
	Roux-en-Y	0.943[0.497,1.793]	0.859	1.129[0.754,1.689]	0.556	1.24[0.857,1.793]	0.254
Surgery time	<270 min	Reference		Reference		Reference	
	≥270 min	1.753[1.054,2.915]	0.03	2.924[2.094,4.084]	<0.001	4.187[3.033,5.780]	<0.001
Intraoperative bleeding	<100 ml	Reference		Reference		Reference	
	≥100 ml	1.878[1.128,3.126]	0.015	4.818[3.410,6.806]	<0.001	7.867[5.559,11.134]	<0.001
Blood transfusion	No	Reference		Reference		Reference	
	Yes	0.526[0.235,1.181]	0.12	1.601[1.069,2.400]	0.022	2.059[1.396,3.036]	<0.001
SPO_2_	≥90%	Reference		Reference		Reference	
	<90%	4.638[2.755,7.806]	<0.001	9.406[6.377,13.875]	<0.001	11.892[7.741,18.270]	<0.001
T-stage	T1~T2	Reference		Reference		Reference	
	T3~T4	1.777[1.068,2.957]	0.027	4.043[2.875,5.685]	<0.001	5.035[3.621,7.002]	<0.001
N-stage	N0	Reference		Reference		Reference	
	N1~N3	2.546[1.524,4.251]	<0.001	3.807[2.711,5.348]	<0.001	5.56[3.984,7.761]	<0.001
PNI	No	Reference		Reference		Reference	
	Yes	4.577[2.474,8.465]	<0.001	3.252[1.950,5.421]	<0.001	2.49[1.490,4.161]	<0.001
Tumor number	<2	Reference		Reference		Reference	
	≥2	1.522[0.885,2.617]	0.129	1.342[0.934,1.928]	0.111	1.106[0.785,1.558]	0.565
Tumor size	<5 cm	Reference		Reference		Reference	
	≥5 cm	1.065[0.581,1.951]	0.838	0.754[0.501,1.134]	0.175	0.65[0.445,0.948]	0.025
CEA level	<5 ng/ml	Reference		Reference		Reference	
	≥5 ng/ml	0.738[0.376,1.448]	0.376	0.918[0.614,1.373]	0.677	0.875[0.603,1.271]	0.483
CA19-9 level	<37 U/mL	Reference		Reference		Reference	
	≥37 U/mL	0.921[0.517,1.642]	0.781	0.899[0.622,1.299]	0.571	0.902[0.642,1.267]	0.552
PCT level	<0.05 ng/ml	Reference		Reference		Reference	
	≥0.05 ng/ml	1.065[0.596,1.902]	0.832	1.043[0.718,1.516]	0.826	0.843[0.593,1.198]	0.341
CRP level	<10 mg/l	Reference		Reference		Reference	
	≥10 mg/l	1.06[0.587,1.916]	0.846	0.857[0.580,1.267]	0.439	0.78[0.543,1.120]	0.179
SAA level	<10 mg/l	Reference		Reference		Reference	
	≥10 mg/l	0.955[0.485,1.883]	0.895	0.799[0.513,1.245]	0.322	0.941[0.631,1.403]	0.765

OR, odds ratio; CI, confidence interval; BMI, body mass index; ASA, The American Society of Anesthesiologists; ALB, albumin; CEA, carcinoembryonic antigen; CA19-9, carbohydrate antigen 19-9; PCT, procalcitonin; CRP, C-reactive protein; SAA, serum amyloid A; NRS2002, nutrition risk screening 2002; CHD, coronary heart disease; COPD, chronic obstructive pulmonary disease; PNI, peripheral nerve invasion; SPO_2_, percutaneous arterial oxygen saturation.

### Model building and evaluation

**Figure 2:**
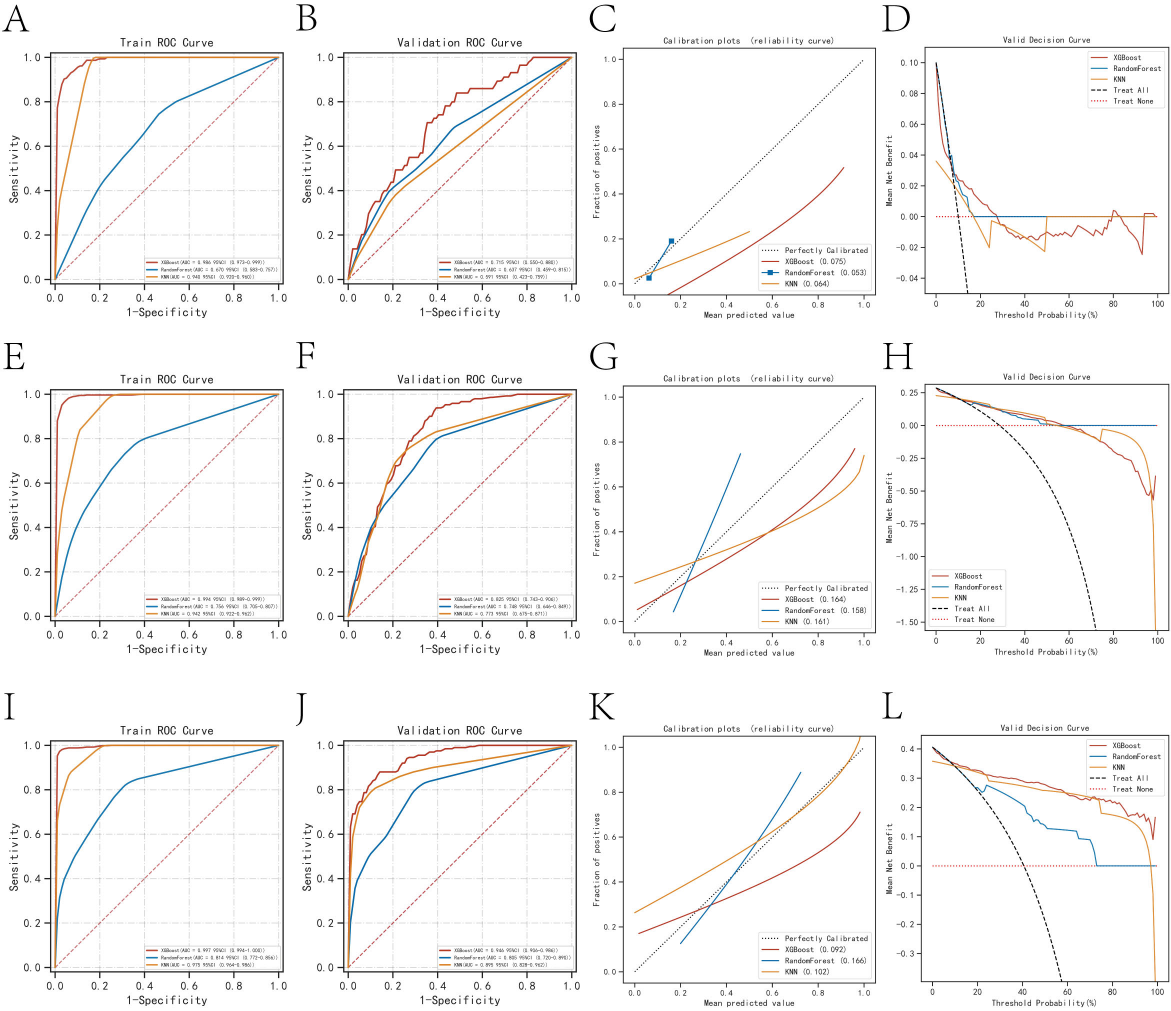
Evaluation of the three models for predicting prognosis. **(A)** ROC curves for the training set of three models predicting patient death at one year. **(B)** ROC curves for the validation set of three models predicting patient death at one year. **(C)** Calibration plots of the three models predicting patient death at one year. **(D)** DCA curves of the three models predicting patient death at one year. **(E)** ROC curves for the training set of three models predicting patient death at three years. **(F)** ROC curves for the validation set of three models predicting patient death at three years. **(G)** Calibration plots of the three models predicting patient death at three years. **(H)** DCA curves of the three models predicting patient death at three years. **(I)** ROC curves for the training set of three models predicting patient death at five years. **(J)** ROC curves for the validation set of three models predicting patient death at five years. **(K)** Calibration plots of the three models predicting patient death at five years. **(L)** DCA curves of the three models predicting patient death at five years.

In the prediction analysis of one-year mortality in patients with anastomotic leakage (AL), the ROC curve results demonstrated that XGBoost achieved an AUC of 0.986 in the training set and 0.715 in the validation set. For three-year mortality prediction, XGBoost yielded an AUC of 0.994 in the training set and 0.825 in the validation set. For five-year mortality prediction, XGBoost attained an AUC of 0.997 in the training set and 0.946 in the validation set. Among the three algorithms evaluated, XGBoost exhibited superior performance across all three outcome indicators (**Table 2**). The calibration curves for all models closely approximated the ideal curves, reflecting strong concordance between predicted and actual outcomes. Additionally, decision curve analysis (DCA) curves indicated that all models provided a net clinical benefit relative to both full treatment and no treatment strategies, suggesting that employing these models for treatment decisions could be advantageous for patients (**Figures 2A–L**).

**Table 2:** Evaluation of the three models.

			AUC (95%CI)	Accuracy (95%CI)	Sensitivity (95%CI)	Specificity (95%CI)	Positive predictive value (95%CI)	Negative predictive value (95%CI)	F1 score (95%CI)	Kappa (95%CI)
One-year mortality	KNN	training set	0.940 (0.920-0.960)	0.932(0.928-0.937)	1.000(1.000-1.000)	0.842(0.827-0.857)	0.469(0.451-0.487)	0.943(0.935-0.950)	0.448(0.433-0.463)	0.395(0.379-0.410)
		validation set	0.591 (0.423-0.759)	0.876(0.853-0.899)	0.355(0.220-0.491)	0.823(0.754-0.892)	0.351(0.198-0.503)	0.921(0.897-0.946)	0.273(0.190-0.357)	0.213(0.101-0.326)
	XGBoost	training set	0.986 (0.973-0.999)	0.930(0.902-0.959)	0.962(0.937-0.986)	0.927(0.895-0.959)	0.304(0.275-0.334)	0.985(0.978-0.991)	0.451(0.425-0.477)	0.365(0.336-0.393)
		validation set	0.715 (0.550-0.880)	0.826(0.791-0.861)	0.826(0.734-0.918)	0.623(0.514-0.732)	0.163(0.066-0.259)	0.928(0.926-0.930)	0.235(0.124-0.346)	0.113(-0.003-0.229)
	RF	training set	0.670 (0.583-0.757)	0.751(0.644-0.858)	0.694(0.499-0.889)	0.605(0.483-0.728)	0.353(0.333-0.373)	0.975(0.972-0.978)	0.491(0.480-0.503)	0.406(0.385-0.428)
		validation set	0.637 (0.459-0.815)	0.736(0.611-0.861)	0.584(0.385-0.782)	0.701(0.564-0.839)	0.065(-0.062-0.191)	0.95(0.941-0.960)	0.252(0.132-0.357)	0.019(-0.168-0.205)
Three-year mortality	KNN	training set	0.942 (0.922-0.962)	0.857(0.840-0.874)	0.943(0.874-1.012)	0.813(0.744-0.881)	0.719(0.635-0.802)	0.845(0.843-0.848)	0.65(0.608-0.691)	0.526(0.471-0.581)
		validation set	0.773 (0.675-0.871)	0.746(0.706-0.786)	0.741(0.627-0.855)	0.767(0.671-0.863)	0.613(0.573-0.653)	0.794(0.729-0.860)	0.504(0.496-0.512)	0.351(0.309-0.393)
	XGBoost	training set	0.994 (0.989-0.999)	0.962(0.956-0.968)	0.970(0.957-0.983)	0.961(0.949-0.973)	0.642(0.621-0.664)	0.911(0.904-0.919)	0.715(0.712-0.719)	0.578(0.566-0.591)
		validation set	0.825 (0.743-0.906)	0.760(0.733-0.787)	0.918(0.868-0.968)	0.693(0.640-0.746)	0.6(0.600-0.600)	0.926(0.915-0.937)	0.698(0.691-0.706)	0.556(0.542-0.569)
	RF	training set	0.756 (0.705-0.807)	0.754(0.721-0.787)	0.749(0.678-0.821)	0.692(0.640-0.743)	0.684(0.681-0.686)	0.907(0.904-0.911)	0.736(0.734-0.737)	0.611(0.609-0.614)
		validation set	0.748 (0.646-0.849)	0.740(0.692-0.788)	0.749(0.648-0.850)	0.700(0.586-0.814)	0.52(0.420-0.620)	0.867(0.836-0.898)	0.573(0.536-0.611)	0.406(0.355-0.457)
Five-year mortality	KNN	training set	0.975 (0.964-0.986)	0.886(0.882-0.891)	0.908(0.861-0.954)	0.907(0.847-0.967)	0.907(0.815-0.999)	0.83(0.768-0.892)	0.782(0.743-0.821)	0.673(0.627-0.719)
		validation set	0.895 (0.828-0.962)	0.858(0.840-0.876)	0.793(0.761-0.825)	0.913(0.877-0.950)	0.89(0.806-0.975)	0.791(0.781-0.802)	0.739(0.645-0.834)	0.6(0.562-0.638)
	XGBoost	training set	0.997 (0.994-1.000)	0.977(0.972-0.982)	0.983(0.978-0.988)	0.977(0.969-0.985)	0.844(0.819-0.870)	0.897(0.888-0.906)	0.843(0.823-0.863)	0.741(0.708-0.774)
		validation set	0.946 (0.906-0.986)	0.866(0.851-0.881)	0.857(0.807-0.907)	0.903(0.867-0.939)	0.784(0.759-0.809)	0.827(0.795-0.858)	0.747(0.691-0.802)	0.597(0.527-0.666)
	RF	training set	0.814 (0.772-0.856)	0.756(0.750-0.763)	0.836(0.797-0.875)	0.681(0.647-0.715)	0.821(0.785-0.856)	0.895(0.875-0.915)	0.829(0.826-0.833)	0.718(0.711-0.724)
		validation set	0.805 (0.720-0.890)	0.738(0.711-0.765)	0.781(0.643-0.919)	0.735(0.646-0.824)	0.878(0.842-0.914)	0.882(0.881-0.883)	0.847(0.808-0.886)	0.749(0.708-0.791)

AUC, area under the curve; RF, random forest; XGBoost, extreme gradient boosting; KNN, k-nearest neighbor algorithm; CI, confidence interval.

**Figure 3:**
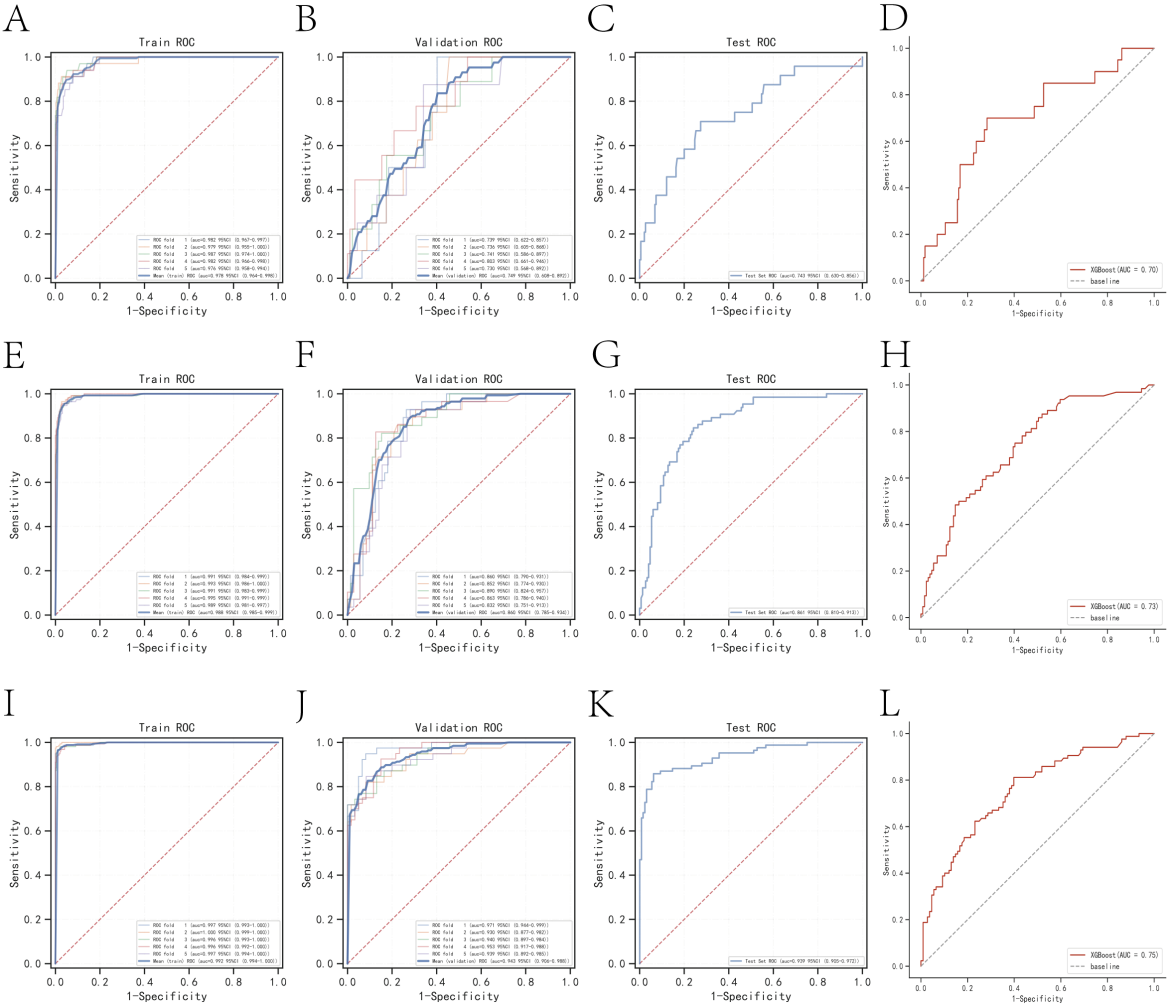
Internal validation of the XGBoost model. **(A)** ROC curve for the training set of the XGBoost model predicting patient death at one year. **(B)** ROC curves for the validation set of the XGBoost model predicting patient death at one year. **(C)** ROC curves for the test set of the XGBoost model predicting patient death at one year. **(D)** External validation of the XGBoost model predicting patient death at one year. **(E)** ROC curves for the training set of the XGBoost model predicting patient death at three years. **(F)** ROC curves for the validation set of the XGBoost model predicting patient death at three years. **(G)** ROC curves for the test set of the XGBoost model predicting patient death at three years. **(H)** External validation of the XGBoost model predicting patient death at three years. **(I)** ROC curves for the training set of the XGBoost model predicting patient death at five years. **(J)** ROC curves for the validation set of the XGBoost model predicting patient death at five years. **(K)** ROC curves for the test set of the XGBoost model predicting patient death at five years. **(L)** External validation of the XGBoost model predicting patient death at five years.

The k-fold cross-validation method was employed to assess the generalization capabilities of the three models. A test set of 214 cases (30.01%) was selected, with the remaining samples used as the training set for 5-fold cross-validation. For the prediction of one-year mortality, XGBoost achieved an AUC of 0.7500 ± 0.0269 in the validation set and 0.7429 in the test set, with an accuracy of 0.8551 (**Figures 3A–C**). In contrast, Random Forest (RF) produced an AUC of 0.6737 ± 0.0736 in the validation set and 0.5842 in the test set, with an accuracy of 0.7477. The k-nearest neighbor (KNN) algorithm yielded an AUC of 0.6122 ± 0.0840 in the validation set and 0.6314 in the test set, with an accuracy of 0.8972.

For the prediction of three-year mortality, XGBoost attained an AUC of 0.8596 ± 0.0189 in the validation set and 0.8613 in the test set, with an accuracy of 0.7991 (**Figures 3E–G**). Conversely, RF exhibited an AUC of 0.7366 ± 0.0313 in the validation set and 0.7549 in the test set, with an accuracy of 0.7056. KNN provided an AUC of 0.8096 ± 0.0202 in the validation set and 0.8102 in the test set, with an accuracy of 0.7850.

The performance of the three models in predicting five-year mortality was as follows: XGBoost achieved an AUC of 0.9465 ± 0.0144 in the validation set and 0.9385 in the test set, with an accuracy of 0.8832 (**Figures 3I–K**). Random Forest (RF) yielded an AUC of 0.7677 ± 0.0634 in the validation set and 0.7977 in the test set, with an accuracy of 0.7523, while k-nearest neighbor (KNN) attained an AUC of 0.9076 ± 0.0197 in the validation set and 0.9207 in the test set, with an accuracy of 0.8458. After a thorough comparison, XGBoost was selected for model construction in this study.

### Model external validation

The AUC values for the external validation set in predicting one-year, three-year, and five-year mortality were 0.70, 0.73, and 0.75, respectively. These values underscore the high accuracy of the prediction model in assessing disease outcomes (**Figures 3D, H, L**).

### Model explanation

**Figure 4:**
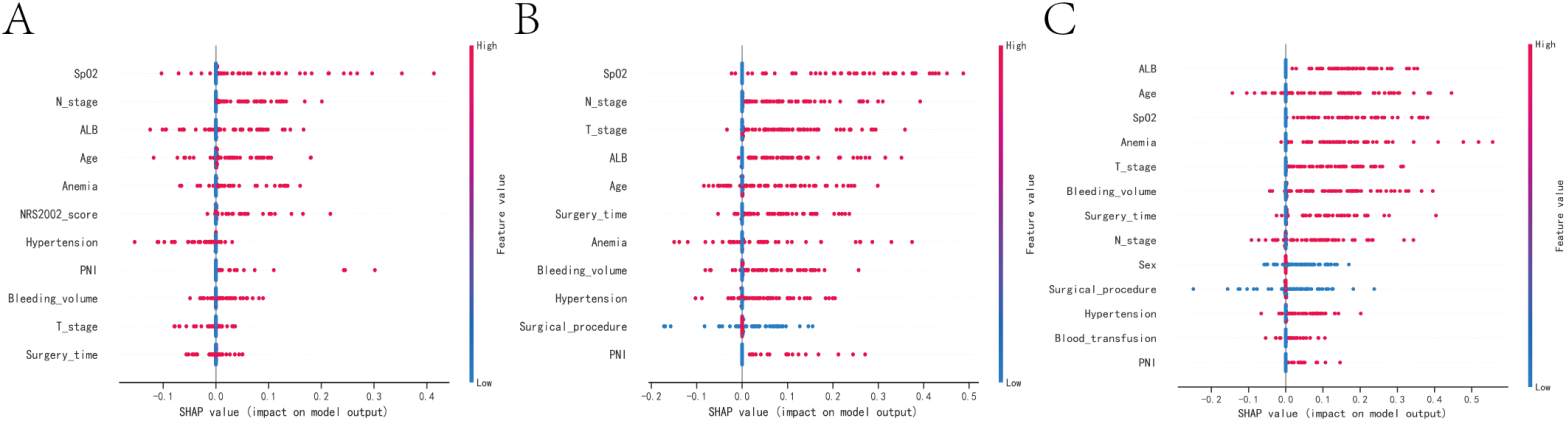
SHAP summary plot. Risk factors are arranged along the y-axis based on their importance, which is given by the mean of their absolute Shapley values. The higher the risk factor is positioned in the plot, the more important it is for the model. **(A)** SHAP summary plot of models predicting patient death at one year. **(B)** SHAP summary plot of models predicting patient death at three years. **(C)** SHAP summary plot of models predicting patient death at five years.

According to the SHAP summary plot results, the risk factors associated with one-year mortality in patients who underwent gastrectomy and developed anastomotic fistula were ranked as follows: low intraoperative SPO_2_, tumor lymph node invasion, hypoproteinemia, advanced age, history of anemia, NRS2002 score, history of hypertension, tumor peripheral nerve invasion (PNI), high intraoperative bleeding, tumors at T3 and T4 stages, and prolonged operative time. The SHAP summary plot results indicated that the risk factors for three-year patient mortality in those with AL after gastrectomy were ranked as follows: lower intraoperative SPO_2_, tumor lymph node invasion, tumors in T3 and T4, hypoproteinemia, advanced age, longer operative time, history of anemia, higher intraoperative bleeding, history of hypertension, surgical approach, and tumor PNI. The SHAP summary plot results indicated that the risk factors for patient mortality at five years in patients with AL after gastrectomy were ranked as follows: hypoproteinemia, advanced age, low intraoperative SPO_2_, history of anemia, tumors in T3 and T4, higher intraoperative bleeding, longer operative time, tumor lymph node invasion, sex, surgical approach, history of hypertension, intraoperative blood transfusion, and tumor PNI (**Figures 4A–C**).

Common factors that contribute to patient mortality over one, three, and five years include advanced age, hypoproteinemia, previous instances of anemia and hypertension, prolonged surgical intervention, heightened intraoperative hemorrhage, suboptimal intraoperative blood oxygen saturation, malignancies in T3 and T4 stages, tumor infiltration in regional lymph nodes, and involvement of peripheral nerves by the tumor.

## Discussion

This study assessed risk prediction models constructed using three machine learning algorithms, with XGBoost emerging as the most accurate. Unlike the RF algorithm, XGBoost employs an adaptive gradient boosting technique that autonomously identifies optimal splitting points and tree depths, thereby enhancing predictive performance. Furthermore, XGBoost effectively mitigates regularization challenges, reducing the risk of overfitting (13, 14). Although the KNN algorithm is lauded for its precision and capacity to minimize overfitting, it demands computationally intensive searches for the K nearest neighbors and distance calculations for each test instance, resulting in significant computational complexity. Moreover, KNN shows diminished robustness and efficiency when dealing with complex scenarios involving numerous features and large datasets (15). In contrast, XGBoost excels in handling multidimensional analyses, reduces computational burden and training time, and offers a feature importance evaluation function that enhances model interpretability. Thus, following a comprehensive comparison of the three algorithms, XGBoost was chosen for developing a model to predict postoperative mortality in patients with AL.

At the outset of the study, we also considered employing other machine learning algorithms for feature selection, such as neural networks, logistic regression, or Bayesian classifiers. However, logistic regression, being a linear model, assumes a linear relationship between features and target variables. In our dataset, the factors influencing mortality in patients with AL encompass numerous complex preoperative and intraoperative variables, potentially involving nonlinear and interactive effects, which logistic regression may not sufficiently capture. Moreover, logistic regression is more sensitive to the distribution of input data and may require extensive feature transformations and preprocessing (e.g., polynomial features, interaction terms) to perform optimally. Instead, we sought algorithms better equipped to handle nonlinear relationships in complex datasets, such as XGBoost and Random Forest, which are more adept at addressing nonlinear problems. While neural networks possess considerable learning capacity, they are susceptible to overfitting due to their large number of parameters, especially when applied to relatively small datasets (e.g., our cohort of 906 patients), where the model may overfit the training set and underperform on the validation set. In contrast, XGBoost and Random Forest are more effective at preventing overfitting and are particularly well-suited for managing the intricacies of clinical data.

Clinical studies often reveal nonlinear effects of various risk factors on patient prognosis, especially in cancer research, where conventional models may struggle to provide accurate predictions. Machine learning, however, excels in training algorithms to recognize complex patterns and adapt to intricate nonlinear relationships, potentially outperforming traditional models in medical research. Liao et al. (16, 17) demonstrated the effectiveness of machine learning algorithms in clinical diagnosis and prognosis, showing that this artificial intelligence technique can accurately predict adverse outcomes in disease progression. This study also leveraged machine learning to construct a predictive model, which can aid clinical decision-makers in accurately identifying high-risk patients and implementing timely interventions to enhance patient prognosis. Moreover, the model can assist medical institutions in the efficient allocation of resources, monitoring vital signs of high-risk patients, and improving the survival rate of gastric cancer patients.

The present study highlighted a higher mortality rate among patients with AL and hypertension. Hypertensive patients often have prolonged high vascular pressure, characterized by reduced elastic fibers and increased collagen fibers in the vessel walls, which elevates the risk of intraoperative bleeding and impairs recovery (18). Afshin (19) observed that hypertensive individuals experience varying degrees of edema in the intestinal wall due to suboptimal cardiovascular system regulation, which undermines the anastomotic site and increases the likelihood of leakage recurrence. Furthermore, fluid compression can lead to insufficient blood supply to the intestinal wall, hindering the healing of the anastomotic segment. Hypertensive patients may also exhibit reduced healing capacity due to their heightened stress levels, which can foster conditions conducive to microbial invasion and proliferation, thereby escalating the risk of inflammatory reactions and adverse prognoses (20, 21). This underscores the need for rigorous monitoring and regular medication during the perioperative period for patients with underlying conditions such as hypertension and diabetes mellitus to manage blood pressure and glucose levels effectively. Clinicians should also offer appropriate counseling to prevent fluctuations in these parameters due to sympathetic excitation, and administer prophylactic antibiotics to avert postoperative infections and complications.

Moreover, research has underscored that the nutritional status of oncology patients is pivotal in determining their prognosis. Patients suffering from anemia and hypoproteinemia exhibit an elevated risk of mortality. Plasma albumin, although a small molecular weight protein, is significantly more abundant than other plasma proteins and plays a critical role in sustaining blood and tissue fluid osmotic pressure. Hypoalbuminemia leads to decreased plasma osmolarity, resulting in intestinal edema and impeding anastomotic healing (21). Additionally, a sufficient blood supply is crucial for the healing of anastomoses, and anemia impairs the delivery of essential nutrients and oxygen, thereby exacerbating the risk of poor outcomes. Preoperative levels of albumin and hemoglobin are also vital for tumor cell immune responses. Patients with compromised nutritional status possess fewer and less active enzymes for antibody synthesis, which heightens the risk of complications such as postoperative infections and AL recurrence (22, 23). Clinicians should regularly assess all nutritional indicators and enhance parenteral nutrition as needed. When patients are able to eat, it is advisable to supplement their diet with high-protein foods.

The findings of this study highlight that extended operative time and heightened intraoperative bleeding are significant risk factors for mortality in patients with postoperative AL. These factors are likely to exacerbate the inflammatory response in patients with compromised physical condition. Additionally, increased apoptosis of macrophages in the internal environment can disrupt normal immune function (24, 25). Such disruptions elevate the risk of complications, including anastomotic infection and AL. Moreover, excessive intraoperative bleeding further diminishes blood supply to the anastomotic segment, impeding its healing. It is advisable for the surgical team to formulate a well-considered surgical plan preoperatively and to collaborate effectively during the procedure to enhance operational efficiency, reduce operative time, and minimize intraoperative bleeding.

Similar to previous studies, the current study has also demonstrated that tumors with higher invasiveness, lymph node metastasis, and PNI are associated with a higher risk of poor prognosis in patients. Such tumors exhibit a high rate of proliferation and low degree of differentiation and possess various protein hydrolases that allow them to degrade the extracellular matrix and basement membrane, facilitating detachment from the primary site. Some of the detached tumor cells invade the surrounding healthy tissues, while others migrate to nearby lymph nodes. The gastric plasma membrane layer, which is abundant in blood vessels, makes it easier for gastric cancer cells to invade the surrounding lymph nodes and cause vascular invasion. Consequently, tumor cells may spread through the portal vein system to the liver, forming distant metastatic foci (26, 27). In contrast, the presence of lymph node metastasis in gastric cancer poses a challenge for achieving complete resection during radical surgery. The abundant lymph node network within the large omentum surrounding the tumor can facilitate ongoing tumor spread following invasion. The extent of tumor spread cannot be identified with the naked eye, complicating the determination of the appropriate scope for surgical resection. Additionally, tumor cells often metastasize to retroperitoneal organs via lymph nodes, with clinical manifestations in patients frequently being subtle and imaging examinations lacking specificity, which exacerbates the mortality risk of gastric cancer patients following surgery (28).

Furthermore, this investigation revealed that SPO_2_ levels below 90% constitute a high-risk factor for mortality in patients with postoperative AL. We hypothesize that the preoperative physical condition of these patients, combined with insufficient oxygen supply during surgery, may impair cardiomyocyte function, potentially compromising cardiac contractility and leading to cardiovascular complications, such as heart failure. Moreover, low SPO_2_ levels may increase blood viscosity, obstruct normal blood flow, and further strain the heart and blood vessels (29). Additionally, compromised metabolic and reparative capacities of the patient’s tissues may result in heightened production and release of cytokines and growth factors, thereby increasing the risk of postoperative AL recurrence (30).

In clinical practice, for patients with AL following gastric cancer surgery, patient monitoring and the use of multiple imaging modalities are commonly employed to diagnose and evaluate the occurrence and severity of the fistula. For instance, patients may be given oral water-soluble contrast agents, followed by X-ray imaging to detect contrast agent extravasation, which indicates the presence of an AL. Alternatively, an enhanced CT scan of the abdomen may be used to help clinicians identify localized leakage around the anastomosis, fluid accumulation, abscesses, or gas buildup in the abdominal cavity. CT scans are particularly valuable in assessing the extent and severity of the AL, especially in complex cases. In certain situations, invasive upper gastrointestinal endoscopy may be utilized to detect fissures or ulcers, guiding subsequent therapeutic interventions.

While these diagnostic tools are crucial for early detection and timely intervention, they present a twofold challenge: on one hand, they contribute to the financial burden on patients; on the other, invasive procedures may exacerbate patient stress and physical discomfort. In regions with limited health insurance coverage or countries where out-of-pocket healthcare expenses are high, these additional costs can impose severe financial strain on patients and their families. This financial pressure may compel some to delay or forgo necessary diagnostic tests and treatments, adversely affecting their prognosis. Moreover, patients who have recently undergone radical gastric cancer surgery are often in a weakened state, and further invasive procedures may trigger stress responses, manifesting as elevated blood pressure, increased heart rate, and heightened pain. Repeated invasive interventions may also increase the risk of secondary complications, such as infection or bleeding, thereby hindering the patient’s recovery.

This study has identified the key high-risk factors influencing patient prognosis. For such high-risk individuals, we will prioritize close monitoring in future clinical practice, utilizing imaging tests as needed to assist with diagnosis and treatment. Conversely, for asymptomatic and low-risk patients, we will employ auxiliary tests more judiciously, thereby alleviating the financial burden on patients and their families while ensuring optimal care.

The present study undertook a thorough evaluation of the model concerning discrimination, calibration, and clinical utility; however, it possesses certain limitations. While a range of risk factors was included, imaging aspects were not considered. Additionally, despite the higher precision of machine learning algorithms, their models are complex and less interpretable. The computational and decision-making processes of the model function within a “black box,” lacking the transparency and intuitiveness of traditional logistic regression models (31, 32). The risk factors identified in this study are not only linked to the development of AL but also serve as crucial determinants of long-term patient prognosis. In future research, we aim to further validate and refine the specific roles of these risk factors across varied clinical contexts by leveraging larger patient cohorts and conducting more detailed analyses in conjunction with treatment regimens, such as chemotherapy duration and dosage. This approach will help to deepen our understanding of how these factors influence outcomes and inform more personalized treatment strategies. Furthermore, as a retrospective study, it is vulnerable to selection bias, distribution bias, and retrospective bias. Therefore, it is crucial to validate the reliability of these findings through subsequent international, multicenter, large-scale studies.

This study demonstrated the exceptional predictive performance of machine learning algorithms such as XGBoost and highlighted its robust interpretability. By utilizing XGBoost models, we confirmed that factors such as advanced age, hypoproteinemia, and a history of anemia were significantly correlated with the prognosis of patients experiencing AL after radical gastric cancer surgery. These models not only accurately forecast patients’ short- and long-term mortality risk but also provide clinicians with a valuable tool to pinpoint key prognostic factors. In future research, we intend to integrate the machine learning models developed in this study into the hospital’s electronic health record (EHR) systems. This integration aims to enhance post-surgical management and safety for gastric cancer patients through automated prediction and real-time risk assessment. By optimizing individualized risk assessment and providing clinicians with more reliable tools for early identification of high-risk patients, we expect to enable timely interventions, ultimately improving patient outcomes.

## Conclusion

TIn conclusion, this study successfully developed an XGBoost-based machine learning model for predicting mortality risk in patients undergoing radical gastrectomy with AL. The model demonstrated robust predictive accuracy and clinical utility, offering surgeons a valuable tool for timely diagnosis. Key predictors of mortality identified included advanced age, hypoproteinemia, a history of anemia, a history of hypertension, prolonged operative duration, substantial intraoperative blood loss, low intraoperative SPO_2_, tumors classified as T3 and T4, lymph node metastasis, and PNI.

## Data Availability

The original contributions presented in the study are included in the article/**Supplementary Material**. Further inquiries can be directed to the corresponding authors.

## Supplementary material

Supplementary Table 1 Raw Data.

Supplementary Table 2 The baseline characteristics of the internal validation set with the external validation set.

Supplementary Table 3 The baseline characteristics of the training set with the test set.
